# Aneurism of Brachial Artery

**Published:** 1879-11

**Authors:** Herman Mynter


### ANEURISM OF THE BRACHIAL ARTERY.

### TREATED WITH LIGATURE OF THE AXILLARY ARTERY AFTER HUNTER'S METHOD.

REPORTED BY HERMAN MYNTER, M. D.

JOSEPH ENGEL, 20 years of age, was sent to me by Dr. Tobie for operative treatment, August 14th, 1879. He is a strong and robust young man, who three months previously was stabbed with a small penknife in the upper and inner part of the right arm. Profuse hemorrhage followed, which was easily arrested by compression; the wound healed in the course of a week. One month afterwards, a little tumor was discovered beneath the cicatrix, as large as a hickory nut, pulsating and painless. It increased gradually in size, until three weeks ago it was as large as a hen's egg. During the last three weeks the growth has been rapid, but the pulsation has disappeared. He has a slight feeling of numbness in the hand, which easily tires with exercise. On examination, a tumor is observed as large as two fists, on the inner side of the arm, beginning two inches below the fossa axillaris. The circumference of the arm over the tumor is 16½ inches, while the same measure on the left arm is but 12 inches. The tumor is 5½ inches long, 7½ inches in the transverse measure. The skin covering the tumor is very thin, of a bluish color, with many small veins. The tumor has some consistency, except on the top, where it is soft and fluctuating over a space as large as a silver dollar.

By slow compression it may be diminished one-third, and by compressing afterwards the brachial artery above the tumor it keeps relaxed, but as soon as the compression is stopped, the tumor is immediately tense again. An aneurismal murmur is heard with the stethoscope over the tumor, vanishing as soon as the artery is compressed above the tumor. The thin brachial artery is felt pulsating above the tumor, but below the tumor no pulsation can be detected in the brachial or radial or ulnar arteries.

August 15th. Operation; under chloroform narcosis; Drs. Tobie, Lothrop, Diehl, and Van Peyma being present and assisting; the axillary artery was ligated about one inch above the tumor; the operation was performed strictly under the carbolic spray. The artery was ligated with carbolized catgut, cut short and left in the wound. The wound was closed with carbolized silk sutures, and covered with antiseptic gauze and salicylic jute. Bottles with hot water were kept around the arm for several days until the circulation was restored. The tumor kept soft for some days, but gradually became more and more solid; the wound healed by first intention without any trace of suppuration.

August 30th. Circumference, fifteen inches; no pulsation in the arteries below the tumor; no murmur; the tumor much smaller and harder; the feeling and the heat of the arm normal; slight numbness at fourth and fifth fingers, decreasing since the operation; Martin's rubber bandage applied over the tumor.

September 6th. Circumference of arm, fourteen and one-half inches; tumor, five inches long, four and one-half inches broad; slight pulsation felt in the radial artery; the arm feels strong and healhy.

September 20th. Circumference, thirteen and one-half; transverse measure, four and one-half; longitudinal measure, five inches.

September 26th. Circumference, thirteen inches; transverse, four inches; longitudinal, four and one-half inches.

October 10th. The tumor is gradually decreasing in size and feels hard, and the patient is at work at his trade as a carpenter.

The interest in this case rests with the antiseptic treatment, and especially with the ligature of catgut, with which it was possible to get union by first intention, and consequently to avoid the danger of secondary hemorrhage, against which no surgeon who uses silk for the ligating of arteries can positively guard himself. Another advantage is that we may disregard the collateral branches, and may ligate, if catgut is used, at any point, inasmuch as the artery, at the point of ligature, is strengthened, instead of weakened, by the new tissue, formed in and around the catgut ligature.

